# Exhaustive search of linear information encoding protein-peptide recognition

**DOI:** 10.1371/journal.pcbi.1005499

**Published:** 2017-04-20

**Authors:** Abdellali Kelil, Benjamin Dubreuil, Emmanuel D. Levy, Stephen W. Michnick

**Affiliations:** 1 Donnelly Centre for Cellular and Biomolecular Research, University of Toronto, Toronto, Ontario, Canada; 2 Department of Biochemistry and Molecular Medicine, University of Montreal, Montreal, Quebec, Canada; 3 Department of Structural Biology, Weizmann Institute of Science, Rehovot, Israel; Fox Chase Cancer Center, UNITED STATES

## Abstract

High-throughput *in vitro* methods have been extensively applied to identify linear information that encodes peptide recognition. However, these methods are limited in number of peptides, sequence variation, and length of peptides that can be explored, and often produce solutions that are not found in the cell. Despite the large number of methods developed to attempt addressing these issues, the exhaustive search of linear information encoding protein-peptide recognition has been so far physically unfeasible. Here, we describe a strategy, called DALEL, for the exhaustive search of linear sequence information encoded in proteins that bind to a common partner. We applied DALEL to explore binding specificity of SH3 domains in the budding yeast *Saccharomyces cerevisiae*. Using only the polypeptide sequences of SH3 domain binding proteins, we succeeded in identifying the majority of known SH3 binding sites previously discovered either *in vitro* or *in vivo*. Moreover, we discovered a number of sites with both non-canonical sequences and distinct properties that may serve ancillary roles in peptide recognition. We compared DALEL to a variety of state-of-the-art algorithms in the blind identification of known binding sites of the human Grb2 SH3 domain. We also benchmarked DALEL on curated biological motifs derived from the ELM database to evaluate the effect of increasing/decreasing the enrichment of the motifs. Our strategy can be applied in conjunction with experimental data of proteins interacting with a common partner to identify binding sites among them. Yet, our strategy can also be applied to any group of proteins of interest to identify enriched linear motifs or to exhaustively explore the space of linear information encoded in a polypeptide sequence. Finally, we have developed a webserver located at http://michnick.bcm.umontreal.ca/dalel, offering user-friendly interface and providing different scenarios utilizing DALEL.

## Author summary

Here we describe the first strategy for the exhaustive search of the linear information encoding protein-peptide recognition; an approach that has previously been physically unfeasible because the combinatorial space of polypeptide sequences is too vast. The search covers the entire space of sequences with no restriction on motif length or composition, and includes all possible combinations of amino acids at distinct positions of each sequence, as well as positions with correlated preferences for amino acids.

This is a *PLOS Computational Biology* Methods paper

## Introduction

The notion that the information encoding molecular recognition could be linearly encoded in peptides has changed the way we have approached the study of protein-protein interactions both experimentally and computationally [1]. Linear information encoding recognition is typically represented using consensus sequences, *i*.*e*. motifs, which define positions and compositions of the residues contributing to both specificity and affinity of recognition [2]. There is a pressing need and great interest in deciphering the linear information encoding recognition for increasingly diverse families of domains involved in all cellular processes [3]. This information is essential to constructing both empirical and quantitative models of biochemical networks [4]. Constructing such models has broad applications to both predicting behaviors of pathways, synthesizing novel pathways to create novel biochemical processes or designing inhibitors of specific cellular processes [5].

For two decades, high-throughput *in vitro* screening methods based on combinatorial peptide chemistry have been utilized to explore the linear amino acid sequence information encoding recognition of linear peptides and to reveal principles that underlie their selective binding to individual domains [6]. These methods involve *in vitro* screening of target domains against peptide libraries to obtain large number of hits, which are then aligned to find positions and compositions of the residues involved in recognition. However, the observation of a protein binding to a peptide *in vitro* does not guarantee binding *in vivo* of the same protein to all proteins encoding the same peptide. There is also the risk of finding peptides that are not found in the cell [4, 7]. Several factors can cause binding to differ between *in vitro* and *in vivo* measurements: *in vitro* detection of domain-peptide interactions are outside of their biological context; that is, binding peptides within a protein are often optimized to bind *in vivo* with concomitant contributions of additional contacts, cooperativity or post-translational modifications [8–10]. There is, thus, always the possibility that contextual information about the binding of a particular linear peptide to a protein is missing from *in vitro* binding data [6]. Moreover, *in vitro* methods are limited in the complexity of libraries, *i*.*e*. number and length of peptides that can be screened, thus limiting the space of linear information that can be explored. For instance, we curated the literature for experimentally validated SH3 domain interactions in yeast, and we found that more than half of the binding sites determined *in vivo* were not currently predicted from *in vitro* experiments [11].

During the past two decades, different classes of methods have been developed in attempts to address these issues. The first class of methods were designed to search specifically for annotated motifs, like those derived from the Eukaryotic Linear Motif (ELM) database [12–16]. For example, Stein and Aloy identified instances of peptide-mediated protein interactions of known 3D structure based on the collection of motifs in the ELM database [16] and explored individual contribution of motifs and context to global binding energy [14], while Mooney *et al*. applied machine learning techniques to predict motifs based on annotated instances from the ELM database [12]. Another class of methods were designed to search for motifs displaying properties previously observed in linear binding peptides, including three-dimensional structures, intrinsic disorder, sequence conservation, and solvent accessibility [17–22]. For example, Stein and Aloy used available structures of known protein-peptide interactions and scanned protein complexes of known 3D structures to identify new peptide-mediated interactions [17]. In another example, Davey *et al*. used the statistical significance of sequence conservation of small stretches of residues within intrinsically disordered regions to identify putative functional motifs [18]. Another class of methods relies on statistical models, such as hidden Markov model, Gibbs sampling and Nested sampling [23–26]. For example, Bailey and Elkan used Gibbs sampling and Expectation Maximization to fit a two-component finite mixture model to describe the sequences of interest, then predicted motifs by fitting the statistical model. In another example, Dogruel *et al*. used a Monte Carlo inference strategy, called Nested Sampling, to build a multi-class sequence background model to find non-motif parts of sequences, then build a set of position-weight matrices to represent motifs overrepresented in the sequences considered [25]. In a different example, Nguyen *et al*. used hidden Markov models to include insertion/deletion/substitution events within protein sequences, then applied the model to identify short linear motifs in the budding yeast *Saccharomyces cerevisiae* [23]. The last class of methods were designed to search overrepresented linear motifs in proteins. For example, Edwards *et al*. used a probabilistic method for identifying overrepresented and evolutionary convergent motifs in proteins [19]. In another example, Kelil *et al*. explored motifs of any length and composition and scored their enrichment using the hypergeometric distribution [27].

Crucial to computational prediction of linear binding sites is the selection of appropriate reference sequences from which statistical inference of discovered binding sites are made. A key advance of the method we describe here are improvements in the selection of such reference sequences. Among existing methods, DILIMOT [21] developed by Neduva and Russell, and FIREPRO [22] developed by Lieber *et al*. score motif enrichment in sequences of interest relative to a set of reference sequences. For DILIMOT, reference sequences are taken arbitrarily from the SWISS-PROT database, while in our method the reference sequences are carefully selected from non-binding negative control proteins. These include the sequences of all proteins that are experimentally shown to bind to one or more members of a family of proteins or protein domains but not to the specific member being tested. Use of such reference sequences should enable us to distinguish between enriched functional motifs and randomly recurring ones. For FIREPRO, reference sequences are also selected from negative control proteins. However, motif enrichment is scored using mutual information, while in our method motif enrichment is scored using the cumulative hypergeometric distribution. This approach offers a significant advantage over the mutual information in that it provides a direct and exact evaluation (*i*.*e*. based on Fisher’s demonstration [28]) of the statistical significance of the enrichment of a motif within the sequences of interest over the reference sequences.

Despite the large number of methods available to date, including all of those cited above, the exhaustive search of motifs in groups of proteins has been so far physically unfeasible. In other words, no method to date allows one to exhaustively explore the entirety of possible linear binding motifs, including flexible residues, *i.e.* positions with preference for multiple amino acids, within a set of proteins of interest and for each motif, calculate a score that evaluates its enrichment relative to a background model.

To address these issues, we have devised a strategy to exhaustively search for linear sequence motifs shared among proteins that form direct interactions with other proteins or folded protein domains, *e*.*g*. from yeast two-hybrid screens. Our strategy can also be applied to any group of proteins of interest to identify enriched linear sequences, or to exhaustively explore the space of linear information. Our method is designed to exhaustively search the entire space of all possible motifs. The search covers all possible sequences of any length and composition within the proteins of interest. Our strategy does not require any prior knowledge about sequence or structural consensus signatures of recognition peptides and can be applied to any group of proteins expected to share sequence motifs (*e*.*g*. because they bind to the same partner). The strategy presented builds on previous work [27] with several notable improvements. DALEL indeed explores preferences for multiple amino acids at each position of a motif, allows combining the background dataset with a dataset of “negative sequences”, and uses a parallel suffix tree to enable the exhaustive search of variations in motifs, which was previously unfeasible due to the associated combinatorial explosion.

To test DALEL we used the SH3 domains of budding yeast *Saccharomyces cerevisiae* as a test system because there are numerous sources of data on SH3 domains’ interactions, both *in vivo* with full-length proteins and *in vitro* with linear peptides. In total, we manually curated 890 protein-protein interactions from the literature, between the 25 yeast SH3 domains and 361 proteins encoding a total of 1073 experimentally verified SH3 binding sites (*i*.*e*. linear peptide segments within the proteins), henceforth called “*known SH3 binding sites*” [11] (S1 Table).

Using DALEL we were able to identify the majority of previously discovered SH3 binding sites. We have also identified previously unreported sites with non-canonical sequences and properties that may bind to SH3 domains in distinct ways or serve indirect ancillary roles in peptide recognition and binding. Finally, in an application of DALEL, we have discovered a remarkable relationship between SH3 domain binding motif thermodynamic, evolutionary properties and functional specificity of the proteins that have given motifs [11]. Application of our approach to other domain-linear peptide interactions may reveal similar relationships, establishing a framework for predicting functional organization of protein interactomes.

## Results

### A generalized definition of binding specificity of proteins for a family of proteins or protein domains

Before describing the methodology for enumerating linear motifs, we first summarize how binding specificity of the motifs for a family of proteins or protein domains is assessed. Our strategy is based on the premise that proteins known to bind to a common target domain are enriched in peptides encoding the linear information necessary to recognize that domain, while all other proteins within a cell or organism do not exhibit such enrichment. In our strategy, we exhaustively enumerate all possible consensus motifs within a set of proteins that bind to one or more types of proteins or protein domains. Our approach is closest to DILIMOT and FIREPRO, and part of its originality comes from the way we define the background model. For each target (one member of a protein or protein domain family), we partition the proteome into three distinct sets. The “*positives*” are proteins that bind to the target. The “*negatives*” are proteins that do not bind to the target but do bind to at least one other member of the same family (*e*.*g*. a different SH3 domain). The “*background*” consists of all other proteins in the proteome that bind neither to the target nor to any of the other members of its family. We calculate then two *p*-values for each motif using the cumulative hypergeometric distribution (Methods): (i) *p*_*NEG*_ quantifies motif enrichment in the positives over the negatives, and (ii) *p*_*BAK*_ quantifies motif enrichment in the positives over the background. In other words, *p*_*NEG*_ reflects specificity of the motifs for the target domain relative to other domains of the same family, while *p*_*BAK*_ reflects specificity for the target domain relative to motifs found in the background. Thus, a motif with strong *p*_*NEG*_ and *p*_*BAK*_ means high specificity for the target domain. The significance of *p*_*NEG*_ and *p*_*BAK*_ are evaluated by calculating their *z*-scores, and the less significant of both *z*-scores is assigned to the motif (Methods). Consequently, when there are not enough (e.g. < 20) well-defined positives and/or negatives, the *z*-scores will inherently be weak and the utility of DALEL will be limited.

### Exhaustive search for linear sequence motifs in proteins

Our strategy exploits suffix trees, which allows for enumerating motifs in sequences in time that is linear with their length and number [29]. Our algorithm searches for all possible motifs comprising any number and combination of wildcards *e*.*g*. X in consensus motif PXXP. Theoretically, the number of possible combinations of wildcards in a motif of length *l* equals 2^*l*^ (*e*.*g*. each position is a wildcard or not, *l*-times), hence, the number of motifs of length *l* in a set of protein sequences is proportional to 2^*l*^, which require a suffix tree with size *O*(2^*l*^) to represent them all. The exponential growth of the suffix tree with motif length makes exhaustive search for motifs unfeasible. For instance, in *S*. *cerevisiae*, the suffix tree required to represent all possible motifs present in SH3 binding proteins rapidly exceeds physical memory (S1 Fig). To address this problem, we devised an algorithm to exhaustively search for motifs by dividing the *O*(2^*l*^) suffix tree into 2^*l*^ smaller suffix trees that can be explored sequentially, resulting in a linear increase in memory usage with motif length (S2 Fig). Such an approach also allows for faster and parallelizable searches.

**Fig 1:**
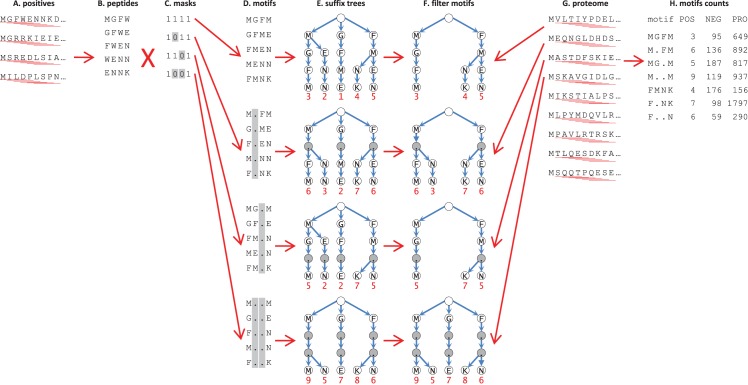
Parallel strategy for exhaustive search of linear motifs in protein sequences. (A) A sliding window is used to enumerate sequence motifs among proteins that bind to a protein or protein domain (positives); (B), the scan is performed for all linear peptides of a specified sequence length. (C) Sequences obtained are passed through a set of masks, each representing one of the possible combinations of wildcard (variable amino acid) positions. (D) Each mask is used to find all possible motifs present in the positives and matching wildcard configuration defined by the masks. (E) A suffix tree is constructed for each set of motifs. (F) The size of each tree is reduced by removing branches corresponding to motifs that occur among the positives less than a specified number of times. (G) Finally, a sliding window is used to scan each protein in the proteome for peptides of the desired length. (H) Peptides are matched to each suffix tree to obtain the number of occurrences of each motif among the negatives (proteins that do not bind to a specified protein or protein domain but bind to one or more members of same family) and the background (all other proteins in the proteome).

The first step of the algorithm consists of using a sliding window to scan the sequences of all positives for linear peptides of a desired length (Fig 1A). The obtained peptides (Fig 1B) are passed through a set of masks, each one representing one of the possible combinations of wildcards (Fig 1C). Each mask is used to find all possible motifs present in the positives and matching wildcard conformation defined by the mask (Fig 1D). Then we build the suffix tree of each set of motifs (Fig 1E). The number of occurrences in the positives of each motif is immediately available after the completion of the construction of the tree, making it possible to further reduce the size of the tree by removing branches corresponding to motifs with occurrences less than a desired minimum (Fig 1F). This optimization contributes significantly to accelerating the next step, which is the slowest in the algorithm. Finally, we use a sliding window to scan each protein in the proteome for peptides of a desired length (Fig 1G) and we match the peptides with each suffix tree to obtain the number of occurrences of each motif in the negatives and the background (Fig 1H).

### Exhaustive search of flexible residues required in peptide recognition

Among peptides known to bind to a common domain, only about one-third of the residues are typically essential for recognition [30, 31], *e*.*g*. “P” in the motif PXXP binding to SH3 domains [32]. Other residue positions can be any (X) or a small number ([…]) of amino acids, *e*.*g*. [RK] in [RK]XXPXXP and PXXPX[RK] motifs binding to diverse SH3 domains [33]. They also often display correlated preferences for amino acids at distinct positions, *i*.*e*. dependence between distinct positions for their amino acids preferences [34], *e*.*g*. [ST] in R[ST][ST]SL peptides binding to Fus1 SH3 domain [35].

**Fig 2:**
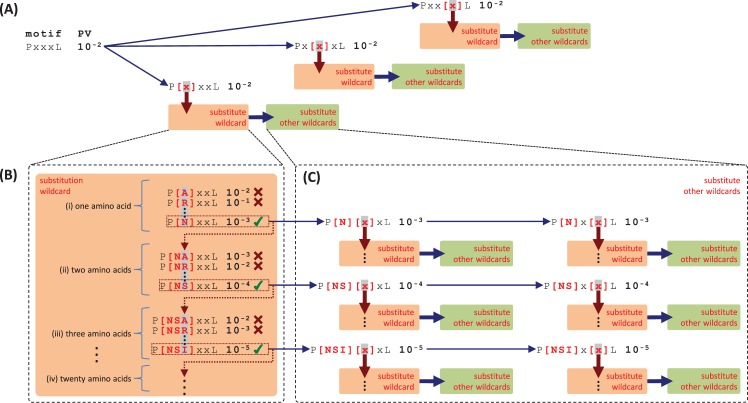
A strategy to exhaustively search for variable residues in linear motifs. The algorithm first exhaustively searches for variants of a motif by substituting each wildcard by all combinations from 1 to 20 amino acids iteratively and test for improvement of *p*-values. (A) Given the motif “PxxxL”, the strategy substitutes all combinations of amino acids at each wildcard (orange boxes). When a substitution at a given wildcard improves the *p*-value, the algorithm switches to substitutions of the other wildcards (green boxes). (B) The wildcard is iteratively substituted by all combinations from 1 to 20 amino acids until there is no further improvement of the *p*-value. (i) At the first step, the wildcard is substituted by each of 20 individual amino acids. Substitutions that improve the *p*-value are retained, *i*.*e*. “P[N]..L”; (ii) for each substitution retained we add, one by one, each of the other amino acids and new substitutions that improve the *p*-values are retained, *i*.*e*. “P[NS]..L”; (iii) step (ii) is repeated for remaining amino acids, *i*.*e*. “P[NSI]..L”. (C) The process described in (B) is simultaneously performed at all other wildcard positions in the motif.

The strategy for exhaustive search for linear consensus motifs consists of finding all possible variants of each motif found in the suffix tree analysis by substituting wildcards by brackets, including all possible combinations of amino acids, *e*.*g*. [IVL] or [DE]. However, exhaustive search for variations in motifs is physically unfeasible because the combinatorial space is too vast. For instance, the number of possible combinations for a single motif with 4 wildcards is on the order of 10^24^. Theoretically, the number of ways of picking *k* amino acids is $C_{k}^{20}=20!/k!(20-k)!$ (*i*.*e*. all combinations of 1, 2, 3, …, and 20 amino acids), and then the number of ways of picking all combinations of 1 to 20 amino acids is $\sum_{k=1}^{20}C_{k}^{20}$ (*e*.*g*. PXXXP has 1 x $C_{k}^{20}$ x $C_{k}^{20}$ x $C_{k}^{20}$ x 1 possible variations). Consequently, the total number of all possible combinations of amino acids for a motif including *n* wildcards is $\prod_{n}\sum_{k=1}^{20}C_{k}^{20}$. Thus, with each additional wildcard the upper bound of the number of possible combinations is multiplied by $\sum_{k=1}^{20}C_{k}^{20}\approx 10^{6}$. For this reason, we devised a strategy where we exhaustively search for all possible variants of each motif that improve its *p*-values (*i*.*e*. *p*_*NEG*_ and/or *p*_*BAK*_), by iteratively substituting each wildcard by all combinations from 1 to 20 amino acids and testing for improvement of the motif *p*-value with each iteration of amino acid substitution (Fig 2).

The substitution of a wildcard starts with a first iteration (Fig 2B) in which a wildcard is substituted by each single amino acid, and substitutions that improve the *p*-value are retained for the next iteration (Fig 2B.i). At the next iteration, for each substitution retained, we add each remaining amino acid one by one and new substitutions that improve *p*-values are retained for the next iteration (Fig 2B.ii). Similarly, we continue at each iteration, to add remaining amino acids to each substitution that improves the *p*-value (Fig 2B.iii). In addition, throughout the iterations, for any substitution that improves the *p*-value, the other wildcards in the motif are substituted in their turn in the same way (Fig 2C). The goal is to exhaustively explore all possible variants of a motif that improve its *p*-values and that present correlated preferences for specific groups of amino acids at distinct positions. Finally, when there is no further improvement in *p*-value with further substitutions, the variable amino acids at a position are retained.

### The method predicts both canonical and unforeseen recognition peptides

**Fig 3:**
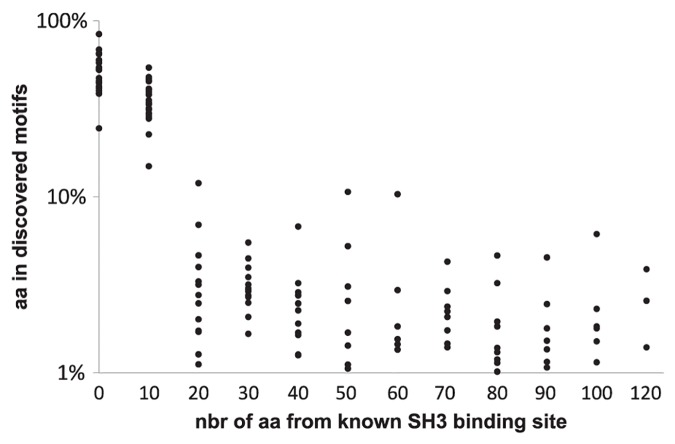
Overlap between discovered motifs and experimentally determined SH3 binding sites. For each SH3 domain, the overlap between discovered motifs and known SH3 binding sites was measured by the distribution of their distances in numbers of amino acid residues (nbr of aa) from the nearest known SH3 domain binding site. The Y-axis represents the frequency of amino acids (aa) that belong to discovered motifs. Along the X-axis, at each position (0, 10, 20, …, 120), each point represents the average frequency of amino acids obtained for a different SH3 domain.

To benchmark the method, we measured how well it could recapitulate known protein binding motifs and unforeseen motifs. Unforeseen motifs are evaluated by comparing general sequence characteristics to those of known SH3 binding sites, *i*.*e*. sequence conservation, intrinsic disorder, solvent accessibility, and predicted binding energy. For each SH3 domain, we thus selected from each of the sequences of positives, those motifs that we discovered with the best *z*-scores and that covered a total length comparable to that of known SH3 binding sites. The procedure yielded 377 motifs from the positives for all SH3 domains except for that of the protein Cdc25, for which the available experimental data was insufficient (S2 Table). We then calculated the overlap between these motifs and known SH3 binding sites. We found that on average, 70% of the amino acids covered by the motifs we discovered were within known SH3 binding sites, and 88% were within 10 amino acids from these sites (Fig 3).

Among the 377 selected motifs, 163 (∼ 43%) matched the canonical SH3 binding consensus motif PXXP, and 52 (∼14%) had sequences longer than 10 amino acids. This latter result is consistent with observations showing that peptides must be longer than the consensus motif for optimal binding affinity and specificity [36]. Further, 307 motifs include positions with variable residues, among which 134 include correlated residues, supporting that cooperativity among residues in binding motifs is common [34]. We found also that on average, ∼35% of the amino acid positions in consensus motifs are wildcards, which is consistent with previous observations on SH3 binding sites [30, 31].

We further analyzed four properties of each of the 377 selected SH3 domain binding motifs, including their binding energy, solvent accessibility, sequence conservation, and intrinsic disorder. We found that 153 of the motifs had similar properties to known SH3 binding sites for the four properties considered, but only 8 had properties that were significantly different (Table 1). We categorized the motifs we discovered into three distinct classes: class I motifs exhibit properties similar to known SH3 binding sites in terms of the four properties and have high overlap (> 80%); class II motifs are highly similar to known SH3 binding sites for the four properties but with less than 20% overlap. These appear to be *bona-fide* SH3 binding sites that may have been missed by previous studies. Finally, class III motifs cover sequences outside of known SH3 binding sites and display markedly different structural and evolutionary conservation properties (Table 1). Nevertheless, motifs in class III exhibit high specificity for their corresponding SH3 domains, suggesting that they may represent non-canonical binding sites or may be indirectly involved in binding. We verified if class III represents coincidental motifs that are enriched in the positives but involved in binding to other domains, as proposed by Edwards *et al*. [37]. For this, we searched whether the motifs we discovered matched known ELM motifs [16]. We searched also instances of these motifs in protein-protein interaction databases (domain-peptide interactions involving binding sites matching our motifs), *e*.*g*. yeastgenome.org, uniprot.org, ncbi.nlm.nih.gov, and thebiogrid.org. We did not find any instance or similarity for the motifs in class III. This result suggests that there exist peptides with distinct properties that may serve ancillary roles in determining SH3 domain binding to proteins. In addition, these motifs may predict interactions that to date could not be determined with existing methods.

**Table 1:** Three classes of discovered motifs. A selection of three classes of discovered motifs. Each row shows **DOM**: SH3 domain for which the selected motif was discovered; **MOTIF**: motifs discovered in this study; **LEN**: motif length; ***p***_***BAK***_: *p*-value scoring motif overrepresentation in positives relative to background (the proteome); ***p***_***NEG***_: *p*-value for motif overrepresentation in positives compared to negatives; ***z*-score**: the *z*-score that corresponds to the least significant of *p*_*BAK*_ and *p*_*NEG*_; **ENR**: binding energy; **SOL**: solvent accessibility; **CON**: sequence conservation; **DIS**: intrinsic disorder; **OVR**: overlap between discovered motifs and SH3 binding motifs of the SH3 domain. Symbols “**+**” and “**-**” mean respectively that, the average of the property (column) for regions in the positives covered by the discovered motif (row) is highly “*similar*” to or “*different*” from the average of the property (column) for known SH3 binding sites; the averages were compared with Student's t-test. We describe in material and methods how each property is obtained.

#	DOM	MOTIF	LEN	*p*_*BAK*_	*p*_*NEG*_	*z*-score	ENR	SOL	CON	DIS	OVR
**CLASS I: motifs covering experimentally identified SH3 binding sites**											
1	RVS167	RPxxP	5	2.93E-14	1.20E-12	5.88	**+**	**+**	**+**	**+**	**93.67%**
2	ABP1	Pxx[PV]xKP	7	5.30E-16	3.53E-13	6.64	**+**	**+**	**+**	**+**	**85.00%**
3	ABP1	PxxxxKPxxL	10	1.19E-12	1.15E-10	5.18	**+**	**+**	**+**	**+**	**92.86%**
4	HSE1	Px[LV]PxK	6	1.92E-21	1.92E-21	9.46	**+**	**+**	**+**	**+**	**82.61%**
5	PIN3	PPL[PS]xR	6	1.12E-08	2.74E-10	4.15	**+**	**+**	**+**	**+**	**100.00%**
6	PIN3	PP[LF]xxR	6	1.13E-07	1.37E-08	3.42	**+**	**+**	**+**	**+**	**91.67%**
7	FUS1	Rx[ST]SL	5	1.77E-10	1.10E-10	4.33	**+**	**+**	**+**	**+**	**82.50%**
8	FUS1	RxxRx[ST]S	7	2.74E-08	1.40E-07	3.29	**+**	**+**	**+**	**+**	**90.00%**
9	BOI1	Px[RK]SxxR	7	7.01E-15	7.01E-15	6.15	**+**	**+**	**+**	**+**	**86.67%**
**CLASS II: motifs covering *de-novo* SH3 binding sites**											
1	YSC84	Px[RMT]xxxxP	8	1.61E-11	1.23E-10	12.13	**+**	**+**	**+**	**+**	**19.63%**
2	YSC84	Px[RP]xxxxP	8	1.67E-12	1.58E-10	12.09	**+**	**+**	**+**	**+**	**19.65%**
3	HSE1	P[QP][PV]L	4	8.83E-09	2.73E-08	9.26	**+**	**+**	**+**	**+**	**18.18%**
4	CYK3	M[EHFP][PSW]K	4	6.68E-07	3.18E-08	9.18	**+**	**+**	**+**	**+**	**0.00%**
5	CYK3	KxP[PT]P	5	6.93E-10	1.06E-07	9.43	**+**	**+**	**+**	**+**	**10.00%**
**CLASS III: motifs covering non-canonical SH3 binding sites or peptides that are indirectly involved in SH3 interactions**											
1	NBP2	M[AP][PS]E	4	6.78E-11	2.97E-11	7.93	**-**	**-**	**-**	**-**	**0.00%**
2	HSE1	F[AEH][FST]L	4	1.34E-07	2.34E-08	5.75	**-**	**-**	**-**	**-**	**0.00%**
3	FUS1	Sxxxx[NDCQGLY]x[NDSY]C	9	3.24E-07	5.01E-07	5.06	**-**	**-**	**-**	**-**	**6.48%**
4	BOI1	L[MPS][NDES]S	4	5.34E-09	2.94E-08	6.45	**-**	**-**	**-**	**-**	**0.00%**
5	BOI1	S[NQE]x[AHLWY]xL	6	3.06E-09	5.76E-09	6.64	**-**	**-**	**-**	**-**	**0.00%**
6	CYK3	I[DELMP]x[NCQLMP]N	5	3.78E-07	5.48E-07	5.01	**-**	**-**	**-**	**-**	**0.00%**
7	CYK3	S[EMPV][LMPWY]VS	5	3.48E-07	7.14E-08	5.38	**-**	**-**	**-**	**-**	**0.00%**

### Prediction of non-standard peptides recognized by Fus1 SH3 domain

**Fig 4:**
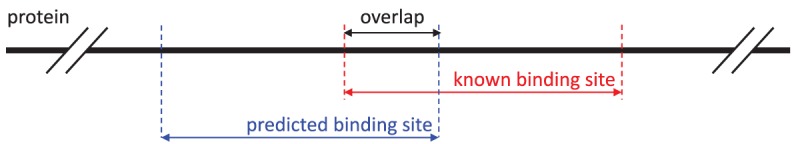
Prediction of non-standard peptides recognized by Fus1 SH3 domain. We compared the motifs we discovered to standard motifs (*i*.*e*. curated from the literature) in the prediction of non-standard binding sites of Fus1 SH3 domain. To this end, we found in the 22 target proteins of Fus1 SH3 domain the peptides belonging to each motif (*i*.*e*. predicted binding sites), then we calculated the overlap with the 25 known binding sites of Fus1 SH3 domain. We found that on average, 70% of the amino acid sequences covered by the motifs we discovered were found within known SH3 binding sites.

Several studies have demonstrated the existence of non-standard SH3 binding sites [38–48]. For instance, the SH3 domain of the yeast protein Fus1 has been shown to recognize peptides belonging to the consensus motif R[ST][ST]SL [35]. To date, 25 SH3 binding sites for the Fus1 SH3 domain, including members of the R[ST][ST]SL motif and others that are not members of any consensus, have been experimentally detected in 22 different proteins. We compared our discovered motifs to standard motifs, those already reported in the literature [4], in the prediction of binding sites recognized by the Fus1 SH3 domain (Fig 4, Table 2).

**Table 2:** Discovered motifs for Fus1 SH3 domain. The table shows for each motif; LEN: motif length; POS: motif frequency in positives; NEG: motif frequency in negatives, PRO: motif frequency in the proteome; the total number of proteins in each of positives, negatives, and proteome is indicated below; *p*_*BAK*_: *p*-value scoring motif overrepresentation in positives relative to the background (the proteome); *p*_*BAK*_: *p*-value scoring motif overrepresentation in the positives relative to the negatives; ENR: binding energy; SOL: solvent accessibility; CON: sequence conservation; DIS: intrinsic disorder; % POS: the total length in sequences of the positives that are covered by discovered motifs; % SH3: the total length of SH3 binding sites that are covered by discovered motifs; (only standard motifs with POS > 0 are shown in the table). Same explanation for symbols “+” and “-” as in Table 1.

#	MOTIF	LEN	POS 22	NEG 571	BAK 5356	*p*_*BAK*_	*p*_*NEG*_	*z*-score	ENR	SOL	CON	DIS	Coverage	
													% POS	% SH3
**Discovered motifs**														
1	R[ST]x[SW]L	5	15	33	134	1E-18	1E-13	9.52	**+**	**+**	**+**	**+**	**0.52%**	**21.08%**
2	Rx[ST]SL	5	13	28	131	5E-15	1E-11	7.87	**+**	**+**	**+**	**+**	**0.45%**	**17.89%**
	**OVERALL**		**22**	**40**	**274**				**+**	**+**	**+**	**+**	**1.26%**	**37.65%**
**Standard motifs**														
1	[RK]xxPxxP (canonical class 1)	7	3	93	464	3E-01	1E+00	0.00	**+**	**+**	**-**	**+**	**0.14%**	**0.00%**
2	PxxPx[RK] (canonical class 2)	6	3	135	500	3E-01	1E+00	0.00	**-**	**+**	**-**	**+**	**0.12%**	**1.81%**
3	Rxx[FLIYM]x[FLIYM]P	7	2	51	253	3E-01	9E-01	0.03	**+**	**+**	**-**	**-**	**0.19%**	**0.00%**
4	Px[ILMVPYAFTR]Px[RKW]	6	2	104	303	4E-01	1E+00	0.00	**+**	**+**	**-**		**0.19%**	**0.00%**
5	[FPLWA]x[WYLMFHP]x[AVLIMFHPR]PxxP	9	2	51	194	2E-01	9E-01	0.03	**-**		**+**	**+**	**0.19%**	**0.00%**
6	[RK]x[AVLIMFHRTP]PxxP	7	2	78	285	3E-01	1E+00	0.00	**+**	**+**	**-**	**+**	**0.19%**	**0.90%**
7	[GP]Px[IVL]xP[FWY]	7	1	2	11	4E-02	2E-01	0.50	**+**	**-**	**-**	**+**	**0.10%**	**0.00%**
8	[KRP]xxxxPxxx[KR]P	11	1	24	111	4E-01	8E-01	0.07	**-**	**+**	**+**	**+**	**0.10%**	**0.00%**
9	[FPLWA]xx[WYLMFHP]x[AVLIMFHP]PxxP	10	1	40	186	5E-01	9E-01	0.03	**-**	**+**	**+**	**+**	**0.10%**	**0.00%**
10	RP[AS]xxxxY	8	2	3	16	2E-03	3E-02	1.09	**-**	**+**	**+**		**0.19%**	**0.00%**
11	R[ST][ST]SL	5	11	5	27	3E-21	1E-11	7.87	**+**	**+**	**+**	**+**	**0.36%**	**16.57%**
	**OVERALL**		**14**	**271**	**1862**				**-**	**-**	**-**	**-**	**1.66%**	**18.98%**

To perform the comparison, we first selected 2 motifs, R[ST]X[SW]L and RX[ST]SL, on the basis of their *z*-scores and because they covered a total length in the 22 target proteins at most equal to the total length of the 25 known binding sites. In fact, the goal here was to limit the number of selected motifs to the minimum, then assess our ability to predict the 25 known SH3 binding sites of FUS1 SH3 domain (Fig 4). The experimentally characterized motifs included R[ST][ST]SL, [RK]XXPXXP and PXXPX[RK] and 15 other motifs determined by phage display screening [4].

We found that the two selected motifs are present in all 22 binding proteins of Fus1 SH3 domain but standard motifs were present in only 14. These two motifs were more statistically significant than all experimentally characterized motifs. Importantly, our motifs matched to 37.65% of the total length of known SH3 binding sites of the Fus1 SH3 domain, while the standard motifs, matched to only 18.98%. Furthermore, the proportion of our motifs found in the entire proteome and negative reference proteins was lower than that of standard motifs. Our motifs were indeed found in only 40 among the 571 negative reference proteins and in 274 among 5356 sequences from the proteome, while the standard motifs were found in 271 of the negatives and 1862 proteins in of the rest of the proteome.

Thus, the motifs we discovered, R[ST]X[SW]L and RX[ST]SL, match the known motif R[ST][ST]SL, but suggest a broader range of substitutions for recognition by the Fus1 SH3 domain, which is consistent with what has been previously proposed [35].

These results suggest that our strategy has helped in redefining a standard motif involved in binding to the Fus1 SH3 domain, without any prior knowledge of their positions or signatures in proteins or what they should look like.

### Prediction of both *in vivo* and *in vitro* experimentally determined SH3 binding sites

**Fig 5:**
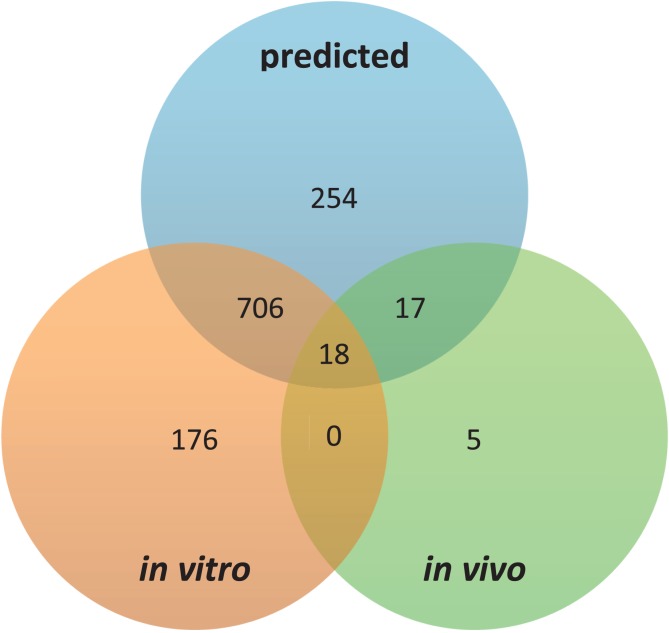
Discovered motifs versus experimentally determined SH3 binding sites both in vitro and in vivo.

The largest number of SH3 domain interactions have been determined in high-throughput screening studies [4, 49, 50]. Among the 922 unique (*i*.*e*. one instance) SH3 binding sites that we found in the literature (Fig 5), 904 (∼98%) were identified *in vitro*, while only 40 (∼ 4%) were identified *in vivo* among which only 18 (∼45%) were also verified *in vitro*. Despite the large spectrum of binding sites discovered *in vitro*, they cover less than half of the peptides that we discovered based on all interactions determined *in vivo*, which suggests that we are still missing a significant number of SH3 binding sites in the yeast proteome. Lack of complete coverage of *in vitro* peptide binding data may be due to, for example, lack of adjacent sequence or of additional interactions that are required for a given peptide within a protein to bind to an SH3 domain [51]. In contrast, the 377 motifs we discovered captured 724 (∼80%) SH3 binding sites among the 904 determined *in vitro* and of among the 40 SH3 binding sites determined *in vivo*, they captured 35 (∼87%). This result highlights the strength of our approach, which discovers equally well both types of sites (Fig 5).

### Prediction of typical properties of peptides recognized by SH3 domains

The degenerate nature of binding peptides makes them hard to detect because they are immersed in a background of mostly irrelevant peptides. For this reason, many approaches reduce the search space using *a priori* knowledge such as canonical motifs. For SH3 binding sites, this includes polyproline peptides that encompass the PXXP core motif or the canonical [RK]XXPXXP and PXXPX[RK] motifs [52, 53]. Additional properties of a given sequence may be taken into account; for example, tertiary structures conforming to predefined structural templates [14, 54, 55] or their presence in regions that are conserved, solvent accessible, or intrinsically disordered [56, 57]. Here we saw that with our strategy, we could accurately detect recognition peptides without using any such knowledge *a priori*. This enabled us to identify peptides such as the structured beta-sheet ubiquitin-like domain of UBI4 recognized by SLA1-3 SH3 domain [48], non-canonical peptides recognized by the Fus1 SH3 domain [35], and additional non-canonical sequences [38–48].

**Fig 6:**
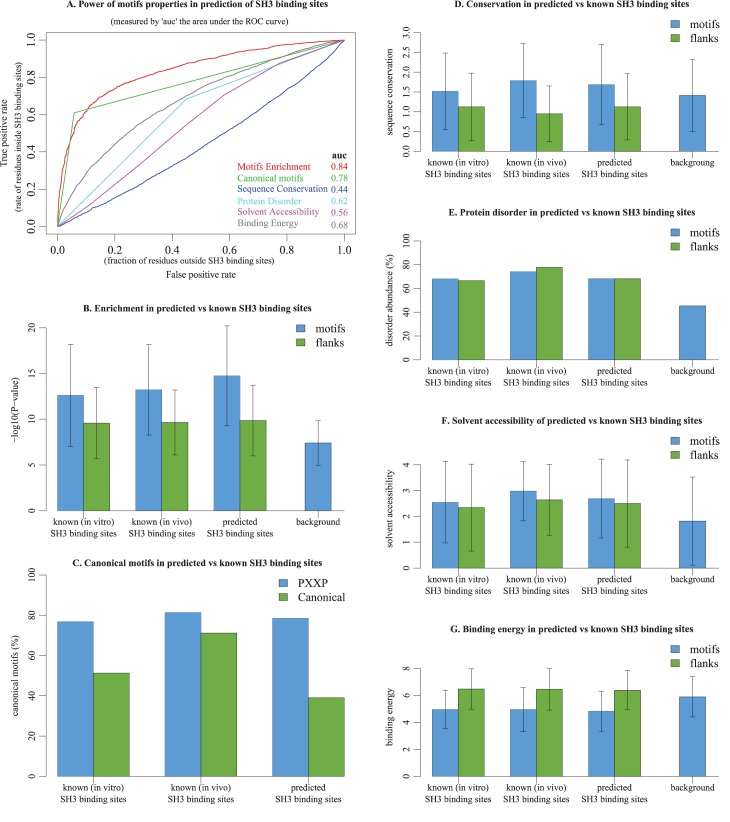
Properties of predicted vs. known SH3 binding sites in contrast with background. A comparison of the different properties of predicted and known (*i*.*e*. experimentally determined) SH3 binding sites, and assessment of the power of each property in the identification of SH3 binding sites among their background. The background is defined as the full length sequences of proteins in which SH3 domain binding sites were discovered. (A). Properties generally observed in SH3 binding sites are used separately as discriminative features for the identification of known SH3 binding sites. The quality of the prediction of each property is evaluated according to the area under the ROC curve (auc). (B). The *P*-value average and standard deviation of the peptides we discovered in known SH3 binding sites in comparison to peptides that we predicted to be SH3 binding sites, compared to peptides we discovered *versus* the background. (C). The abundance of PXXP and canonical peptides (*i*.*e*. [RK]XXPXXP and PXXPX[RK]) in predicted and known SH3 binding sites, compared to the background. (D). The average and standard deviation of sequence conservation in predicted and known SH3 binding sites in contrast to their flanking regions, and compared to their background. (E). The content of protein disorder among predicted and known SH3 binding sites in contrast with their flanking regions and compared to their background. (F). The average and standard deviation of solvent accessibility in predicted and known SH3 binding sites in contrast to their flanking regions and compared to their background. (G). The average and standard deviation of binding energy in predicted and known SH3 binding sites in contrast to their flanking regions and compared to their background. Throughout this figure, the statistics obtained on predicted peptides are calculated based on their occurrences in the proteins.

The absence of any predefined model for the motifs we discovered enabled us to describe, *a posteriori*, the biological properties of corresponding peptides within proteins. We refer here to peptide sequences within proteins that correspond to the 377 discovered motifs predicted to be involved in SH3 binding. Among predicted and known SH3 binding sites, we found that ∼20% of both of them do not belong to the PXXP core motif. Moreover, among known SH3 binding sites, ∼30% of those determined *in vivo* and ∼50% of those determined *in vitro* do not fall into any of the two canonical motifs [RK]XXPXXP and PXXPX[RK] (Fig 6C). Consistent with these results, ∼75% of the SH3 binding sites that we predicted also do not match these canonical motifs.

We also found that >30% of all peptides belonging to our discovered motifs do not fall in intrinsically disordered regions (Fig 6E). In addition, as expected, both predicted and known sites exhibit higher sequence conservation (Fig 6D), solvent accessibility (Fig 6F) and binding energy (Fig 6G) than expected. Interestingly, we found that, in comparison to their flanking regions, like predicted binding sites, known binding sites exhibit stronger contrast in sequence conservation and binding energy and weaker contrast in solvent accessibility, while protein disorder in flanking regions is found to be as high as known binding sites. The contrast between SH3 binding sites and their background is in agreement with known model of SH3 domain recognition [42, 51]. However, these trends vary greatly among binding sites, making it hard to discriminate between binding sites and the rest of the proteins based solely on these general properties. Interestingly, when we used our motifs, we found better contrast than that obtained with general properties between known binding sites and their background (Fig 6B). To assess the power of the different properties in the prediction of SH3 binding sites, we tested each property as a discriminative feature in the identification of known SH3 binding sites (Fig 6A). The prediction results showed that canonical motifs are the best to discriminate SH3 binding sites compared to binding energy, intrinsic disorder, solvent accessibility, and sequence conservation (in decreasing order of their discriminatory power).

Overall, our results highlight three essentials points: (i) SH3 binding sites exhibit common physical properties and sequence conservation, however, these properties are not exclusive to these sites; (ii), although SH3 binding sites exhibit common properties, we have discovered a notable number of sites that have distinct properties; (iii), consequently, as utilized in other methods to search for binding sites in protein sequences, physical properties can be used as a constraint on such searches, but will bias and limit a search and could result in false predictions; for example, of sites that have the right physical properties, but sequences that are not consistent with binding to a domain. In contrast, our exhaustive and unconstrained search strategy should not likely result in any bias, limitations or false-positive results in identifying known or unforeseen linear binding motifs (Fig 6A). This is because our approach takes advantage of the most representative property of the SH3 binding sites: their linear information, *i*.*e*. linear signatures.

These results imply that the number of motifs involved in SH3 domain sequence recognition is larger than generally appreciated. These results highlight that there has been and remains a pressing need for new methods to explore the full complexity of this range of possible binding sites for any given family of protein or peptide binding protein domains. The exhaustive search of linear information in groups of related proteins has been so far physically unfeasible by classic approaches [12–14, 17–26], henceforth this is made possible by the approach we presented here. The approach we applied here, demonstrates prediction performance of SH3 binding sites that is better than approaches that use constrains such as physical properties, sequence conservation, and motifs enrichment. By capturing this large breadth of sequence properties, we were able to discover extensive correlations of physical properties and conservation of motifs and binding specificity, but most strikingly, a correlation of functional and binding specificity that links functional diversity to the chemical and thermodynamic characteristics of SH3 domain-protein interactions [11]. With extensive application of DALEL to other binding domain-protein interactions, we may be able to establish a quantitative framework for predicting functional organization of protein interactomes.

Our strategy should prove an important complement to future efforts to identify linear peptide binding sites within proteins based on simple binary *in vivo* protein-protein interaction measurements, *i*.*e*. a critical step forward in reconstructing protein interaction networks. In addition, while DALEL does not consider motifs with wildcards of variable length, this feature could be developed in the future.

### Comparison of DALEL to other well-established linear motif prediction algorithms

We compared the results of DALEL to those of well-established algorithms on the identification of experimentally determined SH3 binding sites of the protein Grb2. The algorithms tested include iELM [58] that identifies linear binding peptides specifically in the human proteome; MEME [24] detects ungapped linear motifs of fixed-length using finite mixture model; GLAM2 [59] detects gapped linear motifs of variable-length using local sequence alignment allowing insertions and deletions; PRATT [60] finds conserved linear motifs; DRIMUST [61], qPMS7 [26], NESTEDMICA [25], FIRE-PRO [62], DILIMOT [21], SLIMFINDER [63], and MOTIFHOUND [27] find enriched linear motifs in proteins (details in S3 Table).

The Grb2 protein is among recognition domain proteins in human that attract the most interest (http://thebiogrid.org), mostly because of its implication in a large number of protein complexes and canonical cell surface receptor signalling pathways associated with normal cell growth, proliferation and differentiation, and whose component proteins are mutated in a number of cancers (www.proteinatlas.org; www.uniprot.org). The great interest in this protein has helped produce abundance of validated experimental data (http://mint.bio.uniroma2.it), which makes this case “*gold standard*” to evaluate the prediction power of our algorithm, and other algorithms.

The Grb2 protein consists of a central SH2 domain flanked by two SH3 domains (www.rcsb.org). The binding specificity of the Grb2 N-terminal SH3 (N-SH3) domain have been studied in detail and a consensus canonical binding motif “PXXPXR” has been identified [64]. A total of 72 binding sites within 61 different proteins were experimentally determined to bind to the Grb2 SH3-N domain (S4 Table). Here, we compared our algorithm and other algorithms on the identification of the 72 binding sites of the Grb2 N-SH3 domain.

**Fig 7:**
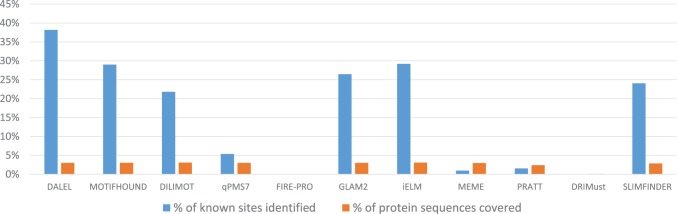
Identification of known binding sites of Grb2 SH3 N-terminal domain. The GRB2 SH3 N-terminal domain is known to bind to 61 proteins, through 72 binding sites that cover 2.45% of their total sequence lengths. We blindly submitted the 61 protein sequences to several algorithms to evaluate their ability to identify these binding sites. We considered the top predicted sites returned for each algorithm, such that they covered at most 3.00% of sequence’s length. For each algorithm, we plot the coverage of the sites identified (red bars), as well as the corresponding coverage of known SH3 binding sites identified (blue bars).

For each algorithm we selected the top scoring predicted sites such that they cover about 3% of the total length of the 61 Grb2 binding proteins, a coverage that is equivalent to that of the 72 known binding sites. The coverage could, however, be smaller than 3% if the algorithm did not return enough sites. For each algorithm, we show the percentage of the total length of the 61 proteins covered by the sites identified (Fig 7, blue bars), as well as the percentage of the total length of known SH3 binding sites covered (Fig 7, orange bars). We found that the sites identified by SLIMFINDER, DILIMOT, GLAM2, iELM, MOTIFHOUND, and DALEL, exhibit the largest overlap with experimentally characterized binding sites, ranging from about 24% to 38%, among which DALEL obtained the highest overlap of 38.19%.

It is interesting to note that iELM performed well, possibly because it integrates properties of binding motifs specifically observed in the human proteome. Also, MEME, that detects ungapped motifs in proteins did not perform well, while its modified version GLAM2 that allows gaps performed much better, because it is better suited for finding degenerate motifs, a property that is present in the binding sites of the Grb2 SH3-N domain. PRATT, however, performed poorly on this example, suggesting that we cannot rely solely on sequence conservation to find linear binding peptides in proteins. In addition, among the algorithms that find enriched linear motifs in proteins only DILIMOT, SLIMFINDER, and MOTIFHOUND obtained satisfactory results.

It is important to highlight the difference between the results obtained by DALEL and MOTIFHOUND, because both find the same motifs. In DALEL, however, the wildcards are degenerated in each motif to find positions with preferences for multiple amino acids, and also positions with correlated preferences. As a result, DALEL identified 38% but MOTIFHOUND only 28% of Grb2 SH3-N binding motifs. This difference proves the discriminative power of our approach because of our strategy for exhaustive search of sequence degeneracy in motifs.

Interestingly, the class **I** canonical motif “PXXPXR” that was reported as recognized by the GRB2 SH3-N domain was identified by DALEL with the significant *z*-score of 4.92 and *p*-value of 1.79x10^-17^. We found the motif “PXXPXR” present in just 29 among the 72 known binding sites of Grb2 SH3-N domain, thus, about 60% did not contain the motif “PXXPXR” (S5 Table). Surprisingly, DALEL, identified the class **II** canonical motif “PXXPXK” with the very significant *z*-score of 5.35 and *p*-value of 2.02x10^-18^. This is surprising because this motif was reported to be recognized by the Grb2 C-terminal SH3 domain [65, 66]. We found this motif present in 22 among the 72 known binding sites of the Grb2 SH3-N domain. More interesting, all of the 22 known binding sites that contain the “PXXPXK” motif did not contain the “PXXPXR” motif. We thus suggest a novel binding preference of the Grb2 SH3-N domain and a possible cross-reactivity between the two SH3 domains of Grb2 protein. In addition, it shows that the binding preference of the Grb2 SH3-N domain is larger and more complex than what was previously reported, because neither the “PXXPXR” or “PXXPXK” motifs explain the binding preference of the Grb2 SH3-N domain to all the 72 known binding sites. DALEL also identified the motif “PXX[PV]XXK” with a significant *z*-score of 5.93 and a *p*-value of 1.08x10^-19^. This motif was present in 42 known binding sites (compared to 22 for “PXXPXK”), This result illustrates the power of our algorithm in allowing preferences for multiple amino acids at specific motif positions.

### Benchmarking the discovery of planted motifs with ambiguous positions

We analyzed the performance of DALEL on the identification of motifs exhibiting ambiguous positions using a benchmark. The benchmark relied on 8 biological motifs from the ELM database [16], which we planted in protein sequences of the *S. cerevisiae* yeast proteome. The advantage of this approach is that there is no simulated data beyond the replacement of amino acids from the original sequence. Thus, sequence features such as tandem-repeats or low complexity regions were preserved.

**Fig 8:**
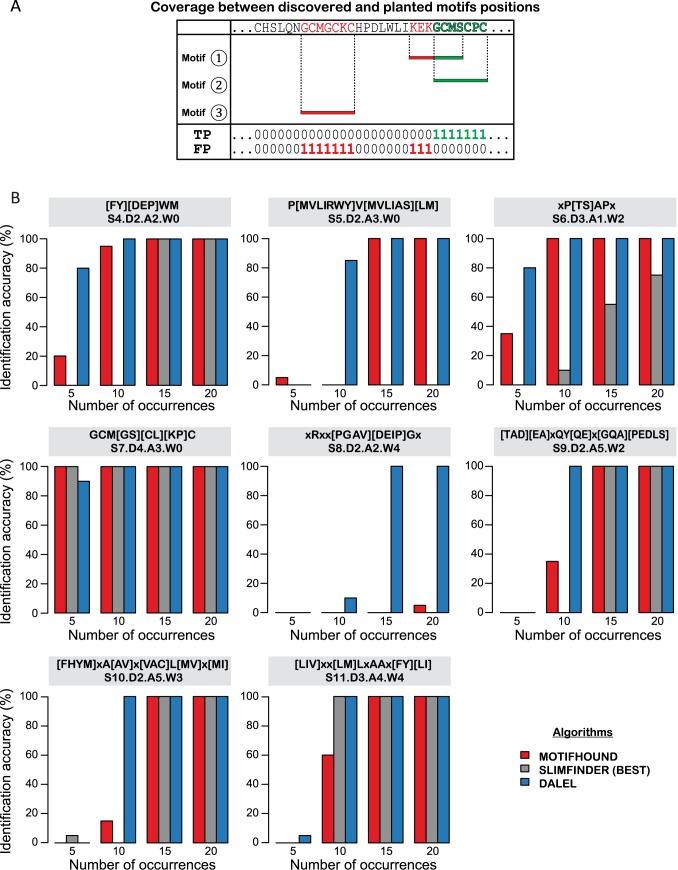
Comparative analysis of three algorithms in the discovery of linear motifs with ambiguous positions. A. For all amino acids covered by a top-ranked motif, we assigned one of the possible prediction outcomes (TP True Positive, FP False Positive) depending on whether the positions matched the positions at which we planted the motifs: TP (number of positions correctly detected), FP (number of positions incorrectly detected). Top-ranked motifs were considered and matched until their sequence coverage (number of TP+FP) reached the number of positions to be discovered. B. The benchmark results for three state-of-the-art algorithms (MotifHound in red, SLiMFinder in grey, DALEL in blue) in the blind discovery of each ELM motif were represented by bar charts. The y-axis reports the global “discovery accuracy” for each number of occurrence (x-axis). The global accuracy is obtained by calculating the fraction of sets in which the planted motif was identified with a precision (TP / (TP+FP)) above 0.7, i.e. an overlap of at least 70% between the positions covered by the top-ranked motifs with respect to the positions of the planted motif. The regular expression of the consensus ELM motif with their key parameters (size S, defined positions D, ambiguous positions A, wildcard positions W) are displayed above each graph.

Degenerate forms of the 8 consensus motifs were planted in 20 protein sets, using 5, 10, 15 or 20 occurrences, with at most one occurrence being inserted per sequence. This gave a total of 640 sets (8 motifs x 20 sets x 4 planted occurrences), in which we subsequently searched for the planted motif. The search in a sequence set was considered successful when the top motif(s) matched the planted sequences with a precision and recall both above 0.7 (Fig 8A). For each motif and each number of occurrences, the fraction of successful searches over twenty sets composed of different sequences was calculated and represented in bar-plots (Fig 8B, Material and Methods).

We compared the performance of DALEL to two algorithms: MotifHound and SLiMFinder. We used MotifHound because it is the closest method to DALEL that does not take into account ambiguous positions, and SLiMFinder because it exhibited the highest accuracy in a previous benchmark [27]. We tested several parameter configurations for SLiMFinder and kept that yielding the best overall results (see Methods). In the best configuration, SLiMFinder successfully identified six of eight motifs present in 15 occurrences and two motifs present in 10 occurrences. MotifHound detected seven out of the eight motifs inserted in 15 occurrences, and three motifs present in 10 occurrences. DALEL, however, showed a significant improvement, as all motifs were correctly identified when 15 occurrences were inserted, and seven were identified when present in only 10 occurrences. Generally, all algorithms had difficulties identifying motifs when only 5 occurrences were planted, although DALEL showed the best results with 3 motifs identified out of the eight investigated (SLiMFinder and MotifHound both only identified one out of the eight).

Our benchmark shows that DALEL performs well in the discovery of motifs with ambiguous positions. Datasets used for this benchmark are available as supplementary material for comparing future improvements of existing algorithms as well as new algorithms.

Despite our efforts to produce bias-free datasets for the benchmark, we cannot ignore the possibility that additional unknown biases may not be addressed, perhaps impeding the performances measured here. Our benchmark largely serves the purpose of testing algorithms that were designed with similar objectives under the same controlled conditions. The artificial nature of the benchmark does, however, have advantages, *e*.*g*. it allows exploring the impact of specific parameters such as overrepresentation of a motif. On the other hand, it also limits our ability to interpret the results in the context of biological motifs discovery. Future work will address these issues.

## Materials and methods

### Calculation of p-values and z-scores

The *p*-values were calculated using the cumulative hypergeometric distribution, which estimates the probability to see at least *k* successes in a sample of size *n* picked from a population of size *N* including a total of *m* successes, and defined as follows:

$$H(k|N,m,n)=\sum_{i=k}^{m}\frac{\left(\begin{array}{c} m\\ i \end{array})(\begin{array}{c} N-m\\ n-i \end{array}\right)}{\left(\begin{array}{c} N\\ n \end{array}\right)}$$ (1)

$$with\;\left(\begin{array}{c} a\\ b \end{array}\right)=\frac{a!}{b!(a!-b!)}$$ (2)

Above, *N* is the size of the proteome, *n* the number of positives, *p* the number of negatives, and by defining a success as a protein comprising the motif; *m*, *k*, and *q* are respectively the number of successes in the proteome, the positives and the negatives. We then calculate *p*_*PRO*_ and *p*_*NEG*_ as follows:

$$p_{PRO}=H(k|N,m,n)\;and\;p_{NEG}=H(k|n+p,k+q,n)$$ (3)

We evaluate the significance of each *p*-value by calculating its *z*-score, which provides the number of standard deviations from the average of the *p*-values distribution, as follows:

$$z_{PRO}=Z(-{log}_{10}(p_{PRO}))\;and\;z_{NEG}=Z(-{log}_{10}(p_{NEG}))$$ (4)

$$with\;Z(x)=\frac{x-\bar{x}}{\sigma (x)}$$ (5)

The transformation here converts the *p*-values from a linear to logarithmic scale, which makes it possible to distinguish between extremely small *p*-values. The *z*-score shows whether a *p*-value is typical or atypical relative to its distribution with respect to its average, $\bar{x}$, and standard deviation, *σ*(*x*).

To summarise, the *p*-values are evaluating the probability of each motif to be enriched in the positives given its presence/absence in the negatives/background, while the z-scores are scoring the enrichment of each motif with respect to all others present in the positives. However, in cases where either the negatives set or the background set is not available, we calculate for each motif one *p*-value, *p*_*NEG*_ or *p*_*BAK*_, and one z-score, *z*_*NEG*_ and *z*_*BAK*_, depending on which set is available. These cases have been considered in the development of DALEL webserver, http://michnick.bcm.umontreal.ca/dalel/Server, to let the user decide which reference set to use, the negatives and/or the background.

### Binding energy

Binding energy was obtained using position-weight scoring matrices developed by Fernandez-Ballester *et al*. [67], and available in the ADAN database [68]. For each SH3 domain in *S. cerevisiae*, the ADAN database provides position-weight matrices predicting the contribution of each amino acid in terms of binding and stability energy between an SH3 domain and a target motif.

### Solvent accessibility

Protein solvent accessibility was obtained by using SABLE version 2 [69], a program used for predicting relative solvent accessibilities of amino acid residues in proteins. In our experiments, only residues with highest confidence level (CI = 9) of solvent accessibility were considered in the analysis.

### Sequence conservation

For an input protein sequence, highly homologous sequences are collected from a proteome reference (*i*.*e*. here fungi proteome) using PSI-BLAST [70] with 35% minimum homology. After that, highly similar sequences among collected homologous are filtered using CD-HIT with 95% maximum homology. After which, remaining homologous sequences including the input protein sequence are aligned using MUSCLE algorithm [71]. Finally, Rate4Site program [72] is applied on the multiple sequence alignment to compute position-specific conservation scores of the input protein sequence across diverse species.

### Intrinsic disorder

Protein disorder was determined using DISOPRED version 2 [73]. Only residues with the highest confidence level of disorder (CI = 9) were considered as disordered.

### Benchmark design for motifs with ambiguous positions

To carry out the benchmark, we planted curated motifs from the ELM database [16] into protein sequences from *S. cerevisiae*, and proceeded with their blind discovery using DALEL, SLiMFinder and MotifHound.

We utilized the proteome of *S*. *cerevisiae* as background in our benchmark. We filtered out homologous sequences by choosing 1 000 sequences of 100 to 500 residues length that showed less than 50% pairwise identity over alignment of at least 50 residues. Among these 1,000 sequences, the average length is 270±117 amino acids and the total number of amino acids is 269,941. We then randomly created 20 sets of 50 sequences within which motifs were planted.

**Fig 9:**
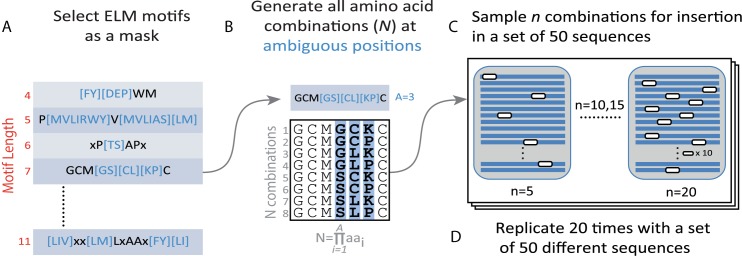
Design of the benchmark datasets. The benchmark is composed of 640 sets of 50 sequences, each set containing a specific planted motif. The planted motifs vary in their size (S4 to S11), number of ambiguous positions (A1 to A6) and number of occurrences (5, 10, 15, 20). We created 20 replicates varying in the motif being planted. Altogether, 160 motifs were created for the benchmark (20 replicates × 8 ELM motifs), resulting in 640 sets of 50 sequences (160 motifs x 4 number of occurrences). A. We first selected 8 motifs from the ELM database with fixed-size of from 4 to 11 residues and with several ambiguous positions. The amino acids within brackets indicate ambiguous positions and x corresponds to wildcard positions with unrestricted amino acid identity. B. In the second step, we derived all N possible combinations of amino acids at ambiguous positions for each motif. In this example, N = 8 unique motifs are generated from a motif containing A = 3 ambiguous positions. C. Finally, each unique motif so obtained is planted in a set of 50 protein sequences selected randomly. In this example, the motif has been inserted 5, 10, 15 or 20 times in the same dataset of 50 sequences. We minimized the level of homology between sequences so that pairwise identity is below 50% for any aligned region of at least 50 residues in length. The white rectangles symbolize the motifs planted, and the blue lines represent protein sequences.

We selected 8 motifs from the ELM database, containing between 4 and 11 residues (S4 to S11) and 1 to 6 ambiguous positions (A1 to A6, *cf*. Table 3). First, for each ELM motif (*i*.*e*. referred as “*regular expression*”, Table 3, Fig 9A), we generated the exhaustive list of amino acid combinations at all ambiguous positions, totalling *N* combinations:

**Table 3:** Properties of selected ELM motifs with ambiguous positions in benchmark design.

Accession	Pattern	S	D	A	W	Description
ELME000170	*[FY][DEP]WM*	4	2	2	0	The YPWM motif confers binding to the PBX homeobox domain
ELME000141	*P[MVLIRWY]V[MVLIAS][LM]*	5	2	3	0	Ligand to interface formed by dimerization of two chromoshadow domains in HP1 proteins.
ELME000099	*xP[TS]APx*	6	3	1	2	PTAP motif binds the N-terminal UEV domain of Tsg101.
ELME000175	*GCM[GS][CL][KP]C*	7	4	3	0	Class 2 Palmitoylation motif
ELME000097	*xRxx[PGAV][DEIP]Gx*	8	2	2	4	The Tankyrase binding motif interacts with the ankyrin repeat domain region in Tankyrase-1 and Tankyrase-2 to facilitate the PARsylation of the target proteins.
ELME000013	*[TAD][EA]xQY[QE]x[GQA][PEDLS]*	9	2	5	2	Members of the non-receptor tyrosine kinase Csk family phosphorylate the C-terminal tyrosine residues of the Src family.
ELME000022	*[FHYM]xA[AV]x[VAC]L[MV]x[MI]*	10	2	5	3	Motif interacts with PAH2 domain in the Sin3 scaffold protein
ELME000021	*[LIV]xx[LM]LxAAx[FY][LI]*	11	3	4	4	Motif interacts with PAH2 domain in the Sin3 scaffold protein

x corresponds to a wildcard character.

S = Size, D = Number of Defined positions, A = Number of Ambiguous positions, W = Number of Wildcard positions.

$$N=\prod_{i=1}^{A}{aa}_{i}$$

In the formulas above, *aa*_*i*_ corresponds to the number of amino acids at the *i*^*th*^ ambiguous position, while *A* corresponds to the number of ambiguous positions (Fig 9B). Thus, each combination samples a unique arrangement of the amino acids inside the square brackets of the corresponding regular expression.

Second, we sampled n degenerate combinations of each motif and planted them randomly either 5, 10, 15, or 20 times in a set of 50 sequences, with at most one motif planted per sequence (Fig 9C). When a motif is planted, wildcard positions take the identity of amino acids already present in the sequence. Each motif was planted into 20 independent sets of 50 sequences (Fig 9D), and this process was carried out for different numbers of occurrences. Altogether, the complete benchmark dataset was composed of 640 sets of 50 sequences (8 ELM motifs x 20 sets of sequences x 4 planted occurrences).

To evaluate the performance of each method, we considered the positions of the sequence dataset matched by the top-ranked (most significant) motifs found by each algorithm (Fig 8A). Therefore, for each amino acid covered by a top-ranked motif, we assigned one of four possible prediction outcome (TP True Positive, FP False Positive, TN True Negative, FN False Negative) depending on whether the positions were matching the positions at which we planted the motifs: TP (number of positions correctly detected), FP (number of positions incorrectly detected), TN (number of incorrect positions not detected), FN (number of correct positions not detected). Note that here, the terms “Positive” and “Negative” used here are not related to the same terms utilized earlier in the manuscript to define binding and non-binding proteins.

Top-ranked motifs were considered and matched until their sequence coverage (number of TP+FP) reached the number of positions to be discovered (TP + FN). We then evaluated the precision of the discovery as: TP / (TP+FP)), which we required to be above 0.7. Thus, we considered a motif to be successfully identified when there was an overlap of at least 70% between the positions covered by the top-ranked motifs with respect to the positions of the planted motif.

We finally calculated a global “discovery accuracy” (Fig 8B) per motif and for each number of occurrence, by the fraction of sets in which the planted motif was successfully identified, *i*.*e*. Number of identifications divided by 20.

## Supporting information

S1 Table We manually curated 890 protein-protein interactions from the literature, between 25 yeast SH3 domains and 361 proteins encoding a total of 1073 experimentally verified SH3 binding sites, *i*.*e*. linear peptide segments within the proteins. Each row in the table gives the interacting SH3 domain protein (columns: **ORF** and **GENE**); the cognate SH3 domain binding protein (columns: **ORF** and **GENE**); the coordinates in the SH3 binding protein of the SH3 binding site (columns: **BEG** and **END**); whether the interaction was identified *in vivo* or *in vitro* (columns: **IN VITRO** and **INVIVO**); and the publication in which the interaction have been identified (column: **PUBMID**).
(XLSX)

S2 Table For each SH3 domain, we selected from each of the sequences of positives, those motifs that we discovered with the best *z*-scores and that covered a total length comparable to that of known SH3 binding sites. The procedure yielded 377 motifs from the positives for all SH3 domains except for that of the protein Cdc25, for which the available experimental data was insufficient. The table gives for each of the 377 motifs (column: **SH3 BINDING MOTIF**); the corresponding SH3 domain (column: **SH3 DOM**); the length of the motif (column: **LEN**); the total number of proteins in the positives, the negatives and the background (columns: **TOT**); and the number of occurrences of the motifs in the positives, the negatives and the background (columns: **NBR**); the *p*-values *p*_*NEG*_ and *p*_*BAK*_, the total length in the proteome covered by the motif (column: **OVR1**), the total length of the SH3 binding sites covered by the motif (column: **OVR2**), the total length of the instances of the motif in the positives covered by the SH3 binding sites (column: **OVR3**), **ENR**: binding energy; **SOL**: solvent accessibility; **CON**: sequence conservation; **DIS**: intrinsic disorder. Symbols “**+**” and “-” mean respectively that, the average of the property (column) for regions in the positives covered by the discovered motif (row) is highly “*similar*” to or “*different*” from the average of the property (column) for known SH3 binding sites; the averages were compared with Student's t-test. We describe in material and methods how each property is obtained.
(XLSX)

S3 Table The table includes the algorithms that have been considered for the identification of known binding sites of GRB2-N domain. For each algorithm we provide the link to the webserver or the implementation. For each algorithm we include the input parameters that whenever a custom value was used. The last column indicates whether the algorithm was tested, or if it was disconnected (was not available at the time we wanted to utilize it) and has not been tested.
(DOCX)

S4 Table We manually curated the literature for 72 binding sites within 61 proteins that have been experimentally determined to bind to the Grb2 SH3-N domain. The table gives in each row, the identification and description of the cognate binding protein (columns: **UPID, GENE**, and **Protein**); the coordinates in the binding protein of the interacting binding site (column: **BEG** and **END**); the method by which the interaction has been determined (column: **Method**); and the publication in which the interaction have been identified (column: **PUBMID**).
(XLSX)

S5 Table The table gives the motif we discovered for each of the 72 experimentally determined binding sites of Grb2 SH3-N domain. Each row gives the cognate interacting protein (column: **Binding protein**); the sequence of the interacting binding site (column: **Binding site**); the coordinates in the interacting protein of the binding site (columns: **BEG** and **END**); the motif we discovered representing the binding site (column **MOT**); the *z*-score and the *p*-value we obtained for the motif we discovered (columns **ZS1** and **PV1**); and whether the binding site fall into any of the two canonical motifs (columns: **PxxPxP** and **PxxPxK**), or both (column: **ALL**), that were reported as recognized by the Grb2 SH3 domain.
(XLSX)

S1 Fig The figure shows that, in *S*. *cerevisiae*, the suffix tree (red curve) required to represent all possible motifs (blue line) present in SH3 binding proteins rapidly exceeds physical memory. To simplify, if we consider that every node in the suffix tree requires a single byte in the physical memory (in reality we need more bytes by node), we will need 10^+13^ bytes in the physical memory to store the suffix tree that represents all possible motifs of length 10, which equals 10^+4^ gigabyte. The size of required physical memory is increasing exponentially with the length.
(EPS)

S2 Fig The figure compares the nonparallel and the parallel strategy in their maximum physical memory requirement to store all possible motifs present in the SH3 binding proteins in *S*. *cerevisiae*. (EPS)

S1 File The compressed file benchmark. 7z contains the 640 protein datasets utilized to benchmark DALEL and the other algorithms. The file README.txt contains a detailed description of the files and directories included.
(7Z) Click here for additional data file.
