# Supplementary material for: Exhaustive search of linear information encoding protein-peptide recognition
Source: PLoS Comput Biol. 2017 Apr 20;13(4):e1005499. doi: 10.1371/journal.pcbi.1005499 (PMC5417721; doi:10.1371/journal.pcbi.1005499)
Supplement: S3 Table — For each algorithm we provide the link to the webserver or the implementation. For each algorithm we include the input parameters that whenever a custom value was used. The last column indicates whether the algorithm was tested, or if it was disconnected (was not available at the time we wanted to utilize it) and has not been tested. (DOCX) [file pcbi.1005499.s003.docx]

Table S3. The algorithms used for the identification of known binding sites of GRB2 SH3-N domain

| Methods | Motif Discovery webservers | Input parameters that we  modified from their default values | Status |
| --- | --- | --- | --- |
| MotifHound | -Proteome=***homos sapiens*** –Motifs length =***3 to 10*** | / | Tested |
| SLiMFinder | <http://bioware.ucd.ie/~compass/biowareweb/Server_pages/slimfinder.php> | / | Tested |
| MEME | <http://meme.nbcr.net/meme/cgi-bin/meme.cgi> | Select the site distribution  **Any number of repetitions**  How many motifs should MEME find?  **10** | Tested |
| FIRE-PRO | [https://iget.c2b2.columbia.edu](https://iget.c2b2.columbia.edu/) | Species: Homo sapiens  **Distribution type: Discrete (0 to n)** | Tested |
| DILIMOT | [http://dilimot.russelllab.org](http://dilimot.russelllab.org/) | Your species: **Homo sapiens**  Maximum S_cons_: **10^-5^** | Tested |
| Pratt | <http://www.ebi.ac.uk/Tools/pfa/pratt/> | Min percentage: **30** | Tested |
| GLAM2 | <http://meme-suite.org> | / | Tested |
| iELM | <http://i.elm.eu.org/search/> | / | Tested |
| DRIMust | <http://drimust.technion.ac.il/> | Statistical significance threshold: **10^-3^** | Tested |
| NestedMica | <http://www.sanger.ac.uk/Software/analysis/nmica/> | / | Disconnected |
| qPMS7 | [http://motifsearch.com](http://motifsearch.com/) | / | Disconnected |
